# Microbiome variation correlates with the insecticide susceptibility in different geographic strains of a significant agricultural pest, *Nilaparvata lugens*

**DOI:** 10.1038/s41522-023-00369-5

**Published:** 2023-01-12

**Authors:** Yunhua Zhang, Tingwei Cai, Maojun Yuan, Zhao Li, Ruoheng Jin, Zhijie Ren, Yao Qin, Chang Yu, Yongfeng Cai, Runhang Shu, Shun He, Jianhong Li, Adam C. N. Wong, Hu Wan

**Affiliations:** 1 State Key Laboratory of Agricultural Microbiology, Huazhong Agricultural University, Wuhan, 430070 China; 2 Hubei Insect Resources Utilization and Sustainable Pest Management Key Laboratory, College of Plant Science and Technology, Huazhong Agricultural University, Wuhan, 430070 China; 3 Department of Entomology and Nematology, University of Florida, Gainesville, Florida 32611 USA

**Keywords:** Microbial ecology, Microbiome

## Abstract

Microbiome-mediated insecticide resistance is an emerging phenomenon found in insect pests. However, microbiome composition can vary by host genotype and environmental factors, but how these variations may be associated with insecticide resistance phenotype remains unclear. In this study, we compared different field and laboratory strains of the brown planthopper *Nilaparvata lugens* in their microbiome composition, transcriptome, and insecticide resistance profiles to identify possible patterns of correlation. Our analysis reveals that the abundances of core bacterial symbionts are significantly correlated with the expression of several host detoxifying genes (especially *NlCYP6ER1*, a key gene previously shown involved in insecticides resistance). The expression levels of these detoxifying genes correlated with *N. lugens* insecticide susceptibility. Furthermore, we have identified several environmental abiotic factors, including temperature, precipitation, latitude, and longitude, as potential predictors of symbiont abundances associated with expression of key detoxifying genes, and correlated with insecticide susceptibility levels of *N. lugens*. These findings provide new insights into how microbiome-environment-host interactions may influence insecticide susceptibility, which will be helpful in guiding targeted microbial-based strategies for insecticide resistance management in the field.

## Introduction

Microbes and their community composition are shaped by a myriad of environmental abiotic and biotic factors, including geographic locations, environmental pH, temperature, precipitation, nutrient availability, and host genotype^1–5^. Changes in the microbiomes mediated by these abiotic factors can significantly affect animal phenotypes^6,7^. As the largest group in the animal kingdom, insects and their associated microbes are constantly responding to environmental variation and fluctuation^8,9^. Growing evidence has demonstrated that many insect phenotypes are the product of microbiome-environment interactions, including insecticide resistance. An example is temperature-dependent changes in microbiome composition that feedback on host insecticide resistance^10–12^.

The brown planthopper, *Nilaparvata lugens* is a significant rice pest in Asia. This pest also has serious resistance problems to multiple insecticides. Having migrated from Southeast Asia to China and other countries every year, this insect is exposed to different insecticides, resulting in geographical differences in resistance profiles^13,14^. Insecticide resistance of *N. lugens* is dependent on the expression of cytochrome P450 (P450) genes, which include *NlCYP6ER1*, a key P450 gene that can mediate resistance to multiple insecticides including imidacloprid, nitenpyram, dinotefuran, thiamethoxam, clothianidin and sulfoxaflor^15–17^. Other genes, including *NlCYP6AY1*, *NlCYP4CE1*, and *NlCYP6CW1*, also play an important role in imidacloprid resistance^18^. Recently, bacterial symbionts have been shown to affect *N. lugens* insecticide resistance by regulating host detoxifying gene expression^19,20^. These findings suggest that insecticide susceptibility variation of *N. lugens* could be determined by the level of detoxifying gene expression regulated by the microbiome.

Although the impact of insecticide exposure as an environmental driver of resistance evolution has been extensively studied, other environmental factors remain underexplored^21–24^. Current literature supports that the environment plays a prominent role in driving microbiome diversification, while the microbiome has been shown to affect insecticide resistance in several agricultural pests. The critical questions we ask in this study are: (1) Do host genetics or microbiome variations correlate with insect differential responses to insecticides? (2) Could specific environmental abiotic factors predict microbiome variation patterns? To this end, we explore the correlation between host genetics background, microbiome composition, gene expression, and insecticide resistance profiles of nine *N. lugens* field strains (FS) collected from different geographical locations and identify significant environmental abiotic factors that may predict these differences.

Our results reveal insecticide susceptibility phenotypes are poorly dependent on host genetics background but associated with multiple bacteria and corresponding detoxifying gene expressions of *N. lugens*. We further demonstrate that several environmental abiotic factors correlated with the variation in abundance of these key microbes. These findings provide new insights that insect host-microbiome interactions may explain the geographical differences in insecticide susceptibility among *N. lugens* strains and lay the foundation for developing targeted microbial-based strategies to manage insecticide resistance.

## Results

### Differences in insecticide susceptibility among the *N. lugens* FS

**Fig. 1:**
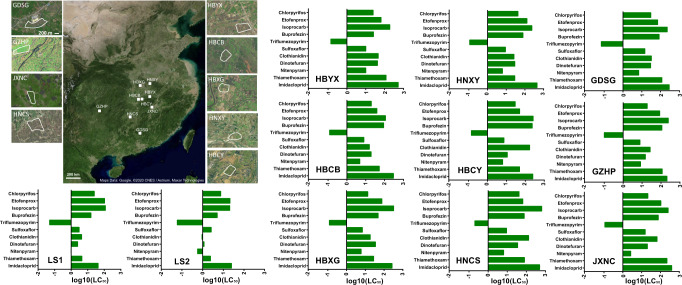
Collection localities and susceptibility level for *Nilaparvata lugens*. The map was downloaded from Google Earth, the area where field strains of *N. lugens* were collected is labeled with white boxes, and the scale bars for small maps were for 200 m. Detailed information on field and laboratory strains of *N. lugens* were shown in Supplementary Table 1. The bar plot shows the susceptibility level [Log10 (LC_50_ value)] of field and laboratory strains of *N. lugens* to 11 insecticides.

**Fig. 2:**
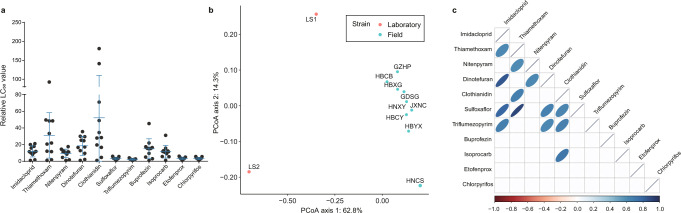
The difference in insecticide susceptibility among 11 strains of *Nilaparvata lugens*. **a** Variation of susceptibility among different insecticides in different strains of *N. lugens*. **b** PCoA based on Bray–Curtis distance of the susceptibilities of laboratory and field strains to insecticides. The color of points indicates the different sources of *N. lugens*. **c** Spearman correlation between LC_50_ of different insecticides among 11 strains, correlations are based on linear Spearman correlation coefficients. The fill color of the ellipses indicates the strength of the correlation (*r*) and whether it is negative (red) or positive (blue). A higher absolute value will show a narrower area of ellipses down to a line (*r* = 1). Only significant correlations with *P* < 0.05 are shown. If the correlation is not significant, the box was left white.

To investigate the pattern of insecticide susceptibility among field *N. lugens*, the susceptibilities of nine FS collected from different geographic locations to 11 insecticides were measured. Two laboratory strains were also included (Fig. 1). The LC_50_ results indicate that the insecticide susceptibility of *N. lugens* varies by strain (Fig. 2a, Supplementary Table 4–14). Specifically, susceptibility to clothianidin had the highest inter-strain variability, whereas triflumezopyrim had the lowest. The susceptibility to neonicotinoid insecticides (imidacloprid, thiamethoxam, nitenpyram, dinotefuran, and clothianidin), buprofezin, and isoprocarb have greater variations as compared to the other 4 insecticides (Fig. 2a, Supplementary Table 4–14). The susceptibility profiles of lab strains (LS) were significantly separated from FS in PCoA1, and the results also showed that eight out of nine FS are similar but not the HNCS (PERMANOVA *r* = 0.557 *P* = 0.021, Fig. 2b). Spearman-based correlation was used to verify cross-resistance between the different insecticides, through which we found there were positive associations between insecticides acting on the nicotinic acetylcholine receptors (Fig. 2c). Imidacloprid showed a serious cross-resistance problem with four other insecticides (thiamethoxam, dinotefuran, sulfoxaflor, and triflumezopyrim), which implies similar mechanisms underlying insecticide resistance among the FS for these insecticides.

### Insecticide susceptibility differences correspond to detoxifying gene expression in *N. lugens*

**Fig. 3:**
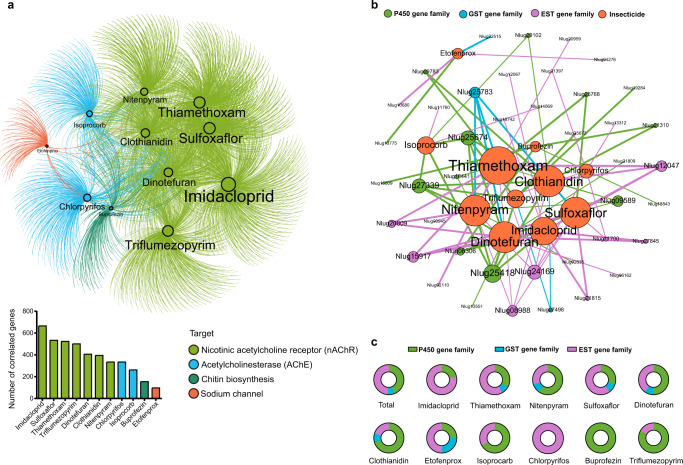
Correlation networks of LC_50_ values and genes expression levels. **a** Interaction network of LC_50_ values and whole transcriptome genes expression levels, the circles are reflected insecticide susceptibility, and their size reflected the number of lines connected to them (more lines result in bigger node sizes), edges indicated the correlation between its connected nodes and nodes represented genes expression levels, while the color indicated a different target of insecticides. Barplot in the lower right corner described the number of genes correlated with each insecticide. **b** Interaction network of LC_50_ value and detoxifying genes expression levels. The color of nodes and edges represented the type of detoxifying genes. **c** Proportion of different detoxifying genes associated with each insecticide which showed in the network. In both networks, the size of the node with labels indicates the number of genes associated with it. The line between the two circles indicates a significant correlation (*P* < 0.05). The color of the circle represented the type of detoxifying genes. Detailed information is shown in Supplementary Data 2.

To explore the mechanisms underlying the differences in insecticide susceptibility among the *N. lugens* strains, we analyzed the transcriptomes of all the FS and LS (Supplementary Table 15 and Supplementary Data 1), as insecticide susceptibility variation is usually dependent on detoxifying gene expression level^25^. There are significant differences among the transcriptomes of the 11 strains (PERMANOVA, *r* = 0.445, *P* = 0.006, Supplementary Figure 1) but not between the FS and LS (PERMANOVA, *r* = 0.037, *P* = 0.266, Supplementary Figure 1). Intriguingly, dense networks were obtained when we analyzed the correlation between *N. lugens* transcript abundances and susceptibilities to individual insecticides (Fig. 3a), demonstrating the intimate connections between host insecticide susceptibility and gene expression. Genes related to insecticide susceptibility of *N. lugens* were shared frequently (926, 35.67%), especially among insecticides in the same target group (Fig. 3a and Supplementary Data 2). A majority of the genes correlated with the LC_50_ of imidacloprid (666), dominated by negative correlations (468, 70.27%). Insecticides that target the acetylcholine receptor have a greater number of associated genes than chitin synthesis inhibitors (buprofezin) or etofenprox that targets neuron sodium channels (Fig. 3a and Supplementary Data 2). We performed correlation analysis on gene families of three major detoxifying enzymes, the P450 monooxygenase, glutathione *S*-transferase (GST), and esterase (EST), which are known to affect insecticide susceptibilities. There are 41 detoxifying genes (18 of P450, 3 of GST, and 20 of EST) in total that are correlated with at least one insecticide (Fig. 3a, c). Notably, the expression of *CYP6ER1* (includes several variant transcripts), a key P450 gene, is positively correlated with LC_50_ of 7 insecticides to *N. lugens* including, imidacloprid, nitenpyram, dinotefuran, clothianidin, triflumezopyrim, buprofezin, and isoprocarb) (Fig. 3b, c and Supplementary Data 2). These results indicate that differences in insecticide susceptibility among the *N. lugens* strains can be explained by the differential expression of key detoxifying genes.

### Microbiome variations correlate with detoxifying gene expression

**Fig. 4:**
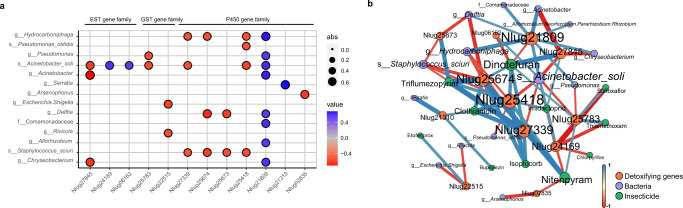
Microbiome as a potential shaper in detoxifying genes expression of *Nilaparvata lugens*. **a** Correlations between detoxifying genes expression and core bacteria abundance. Detoxifying genes are classified into three intervals by function (P450, GST, and EST, respectively). The size of the node showed the absolute value of correlation, blue means positive correlation, and red means negative correlation. Only genes associated with at least one core microorganism were shown. **b** Correlation network between key detoxifying genes with insecticide susceptibility and microbial abundance. Correlations are based on Spearman correlation coefficients and the node size is positively proportional to its degree, which indicates the number of correlated factors. The line between the two circles indicates a significant correlation (*P* < 0.05), the blue edge line indicates a positive correlation, and the red edge indicates a negative correlation. The thickness of each edge is proportional to the correlation coefficient. Detailed information is shown in Supplementary Data 5.

The microbiome can modulate insecticide resistance by influencing the host’s detoxifying gene expression, as shown in various insect pests, including *N. lugens*^11,20,26^. This leads us to hypothesize that the different insecticide susceptibility and detoxifying gene expression profiles among the *N. lugens* strains may be attributed to microbiome differences. A total of 372 bacterial and 1732 fungal taxa were identified across the 11 *N. lugens* strains by 16S rRNA and ITS amplicons sequencing, respectively (Supplementary Table 16, Supplementary Data 3 and 4). The dominant bacteria include *Wolbachia, Arsenophonus, Acinetobacter_rhizosphaerae*, and *Staphylococcus_sciuri*, which comprised over 80% of the reads, and the genus *Hirsutella* dominated the fungal communities (Supplementary Fig. 2a, b). Inter-strain variation of bacterial and fungi communities based on Bray-Curtis dissimilarity is highly significant [(Supplementary Fig. 2c, d) PERMANOVA, *r* = 0.423, *P* = 0.001 for bacteria; PERMANOVA, *r* = 0.475, *P* = 0.001 for fungi], and also between FS and LS [(Supplementary Fig. 2c, d) PERMANOVA, *r* = 0.055, *P* = 0.015 for bacteria; PERMANOVA, *r* = 0.12, *P* = 0.001 for fungi)]. Subsequently, we analyzed the correlation between gene transcript and microbial taxon abundances, focusing on taxa with relative abundance greater than 0.05% and existing in more than 60% of the samples, i.e., 11 fungal and 33 bacterial genera, which we defined as “core” (Supplementary Table 17). In the analysis including all strains, we examined the correlation between the expression of detoxifying genes and the microbial taxa, in which 7 of the P450, 2 of the GST, and 3 of the EST genes correlated with specific bacterial taxon abundances, while no gene correlated with specific fungal taxon abundances (Fig. 4a and Supplementary Data 5). Among these detoxifying genes, five *NlCYP6ER1s* (Nlug21310, Nlug25418, Nlug25673, Nlug25674, and Nlug27339) have been previously shown to be involved in *N. lugens* insecticide resistance (Supplementary Table 18); their expressions significantly correlated with the abundances of six bacteria (Fig. 4a). Notably, the expression of *NlCYP6AX1* shows a strong negative correlation with the abundance of genus *Arsenophonus*, a symbiont involved in host insecticide resistance (Fig. 4b)^20^. These results suggest that the abundances of certain bacteria are potential markers for insecticide susceptibility of *N. lugens* FS. The significant detoxifying gene-microbiome correlations also imply these genes are important nodes connecting variations of the microbiome and host responses to insecticides.

### Variations in microbiome diversity and composition among the *N. lugens* FS are significantly associated with specific environmental abiotic factors

**Fig. 5:**
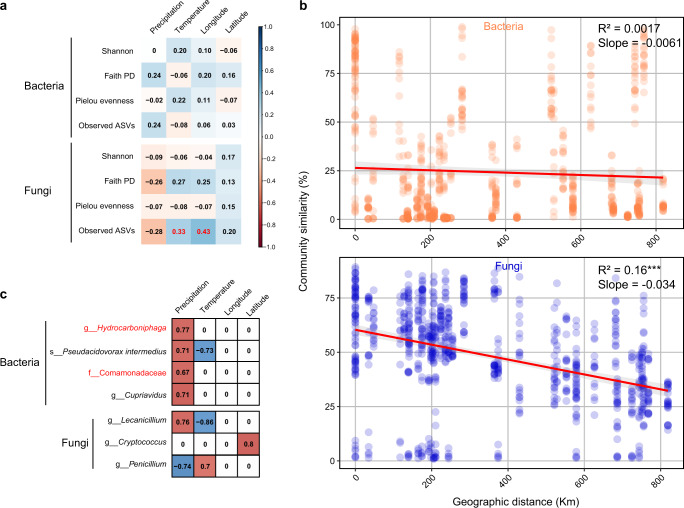
Correlations between core microbial abundance with environmental abiotic factors among field strains of *Nilaparvata lugens*. **a** Heatmap of correlations between different α-diversity indexes and environmental factors. The background color indicates the strength of the correlation (*r*) and whether it is negative (red) or positive (blue). All the correlations were shown but only red numbers represent significant correlation (*P* < 0.05). **b** Distance–decay curves of similarity for symbionts communities. Red lines denote linear regression. Asterisks represent the significance of correlation (***P < 0.0001). **c** Correlations are based on Spearman correlation coefficients; blue means positive correlation, and red means negative correlation. No significant correlation (*P* > 0.05) was shown in white color. Microbial labels in red color were key microbial in Fig. 4b.

**Fig. 6:**
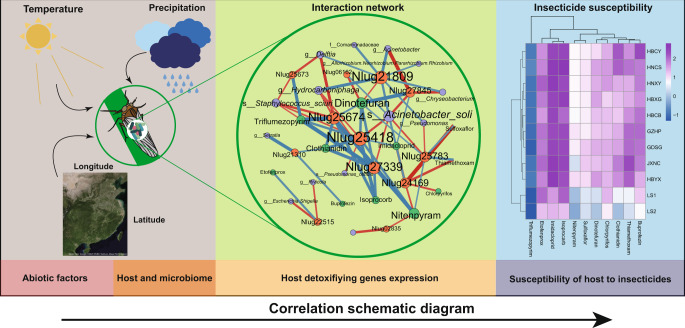
The diagram of environmental abiotic factors associated with microbiome variation correlated with detoxifying gene expression led to the difference in insecticide susceptibility among field *Nilaparvata lugens* strains. The map was downloaded from Google Earth.

So far, our data show that the different field *N. lugens* strains varied in insecticide susceptibility, gene expression, and microbiome profiles, and we have identified specific correlation patterns. To further investigate how host genetics and environmental abiotic factors may contribute to these correlation patterns, we ran additional correlation analyses with respect to the core microbiome. Core microbiome diversity based on Bray–Curtis dissimilarity matrices were weakly correlated to host genetic background (*P* = 0.051 and 0.053 for bacteria and fungi, respectively; Supplementary Fig. 3 and Supplementary Table 19). To disentangle the association between environmental parameters and microbiome diversity of *N. lugens*, we explored the correlation between microbiome α-diversity indexes and various climate and location parameters of the *N. lugens* FS (Fig. 5a). The results showed that observed ASVs of fungi, but not bacteria, were significantly correlated with temperature (*P* = 0.037) and longitude (*P* = 0.0052) (Fig. 5a). We subsequently estimated the distance decay of symbionts community similarity. Significant distance–decay relationships were also observed in fungi (slope = −0.034, *P* < 0.0001) but not in bacteria (slope = -0.0061, *P* = 0.1215), which suggests the fungal community similarity decreased with increasing geographic distance (Fig. 5b). Importantly, among the core microbiome taxa, the abundance of 3 fungi and 4 bacterial genera are significantly correlated with precipitation, temperature and/or latitude (Fig. 5c). Among these microbiome taxa, two bacterial symbionts (g__*Hydrocarboniphaga* and f__*Comamonadaceae*) (Fig. 5c) were significantly correlated with detoxifying genes expression (Fig. 4b), which have been shown to affect *N. lugens* insecticide susceptibility. Together, our results suggest environment may have a stronger effect than the host genotype on *N. lugens* microbiome composition. These abiotic factors may ultimately lead to differences in insecticide susceptibility among *N. lugens* FS by effects on the microbiome and detoxification metabolism (Fig. 6).

## Discussion

The present study investigates the underlying drivers and mechanisms of the inter-strain variability of *N. lugens* insecticide susceptibility in FS. Our results suggest that environmental abiotic factors may be better predictors than host genotypes for microbiome variation. We also revealed host gene-microbiome interaction networks and identified gene markers for insect response to each of the nine insecticides tested, based on correlations. These findings signify that insecticide resistance phenotype among the field *N. lugens* strains are the product of interactions between the environment, microbiome, and host detoxification metabolism (Fig. 6).

Differences in insecticide susceptibility among field strains observed in our study agreed with previous reports^13,14^. The higher basal expression of detoxifying genes could be a consequence of chronic insecticide exposure^27^. Therefore, it is plausible that the expression patterns of detoxifying genes reflect insecticide usage in different locations, and some of these genes may be used as diagnostic markers for pest resistance prediction, and guide resistance governance strategies^28^. In our previous study, the *N. lugens* symbionts have causal effects on host detoxification metabolism and insecticide resistance^11^. The correlation we identified from field *N. lugen* strains, when taken together with the accumulated knowledge from our previous studies, favors our conclusion that microbiome variations are likely contributing factors to insecticide susceptibility rather than consequences of insecticide exposure.

Our study suggests that the host transcriptome correlates with the microbiome. Microbiome diversification among the strains could be the by-product of varied insecticide exposure, it can also be shaped by other abiotic factors, including temperature and humidity^29,30^. One example is elevated temperatures causing declines in insect–microbe symbiosis and symbiont-dependent insecticide resistance^11^. Based on our findings that the abundances of certain microbiome taxa significantly correlated with climate and geographical factors, more research shall pay attention to how changing environment (e.g., climate change) may impact microbiome diversity that could ultimately impact insecticide resistance phenotype of insect pests^31,32^.

*N. lugens* harbors two strains (S-type and N-type) of *Arsenophonus*. Replacing S–type with N-type was previously shown to decrease insecticide resistance due to the downregulation of the P450 genes *CYP6AY1* and *UGT*^20^. In this study, we also revealed a positive correlation between *Arsenophonus* abundance and the expression of the key P450 gene, *NlCYP6AX1*. The expression levels of this gene may correlate with resistance to nitenpyram in *N. lugens*, suggesting *Arsenophonus* could serve as a potential target to combat *N. lugens* insecticide resistance^25^.

In our recent laboratory study, we observed that *Wolbachia* was a key player in *N. lugens* insecticide resistance^11^. However, we do not observe any correlation between *Wolbachia* and detoxifying gene expression in field *N. lugens* strains. This may be because the influence of *Wolbachia* on host insecticide resistance is unique to specific *Wolbachia* strains, or particular host genotype background^33–35^.

Our work also points to an “insecticide-resistant microbiome” concept. If resistance can be predicted by the presence or abundance of particular taxa, microbiome variation may be regarded as a characteristic contributing to differences in insecticide susceptibility among the *N. lugens* FS. This characteristic may be disseminated in the field as the microbiome is transmissible^36,37^. In fact, some key symbionts, such as *Arsenophonus*, have been confirmed to spread horizontally among field populations^38^. Thus, the dissemination of insecticide resistance may result from microbiome transmission. This may explain why some insecticides have been banned for years, yet resistance levels remain high in the insects (such as *N. lugens* to imidacloprid)^25^.

In conclusion, our results reveal that microbiome variation in different geographic strains correlates with the insecticide susceptibility of *N. lugens*. However, these correlations alone do not deduce any causal relationship, more experiments will be needed to establish causality. In addition to the microbiome, variations of genetic background among these strains may contribute to insecticide susceptibility differences but the effects of genetic background were not further evaluated in this study. It is also important to note that our analyses were based on *N. lugens* samples collected in one year. We expect there would be variations across years that justify our future work to sample multiple years. Nevertheless, our findings provide new insights into the heterogeneity of insecticide resistance in field insect populations in the context of potential microbiome-host-environment relationships.

## Methods

### Insect strains

*N. lugens* FS were collected from rice paddy fields from nine locations in six provinces in China in 2019 (Fig. 1 and Supplementary Table 1). Laboratory strain 1 (LS1) was collected from a single rice field at Huazhong Agricultural University in Wuhan, China, in 2008 (Supplementary Table 1). The laboratory strain 2 (LS2) was obtained from Zhejiang Chemical Industrial Group Co. Ltd (Hangzhou, Zhejiang, China). It was originally collected in 1995 from a rice paddy field near Hangzhou, Zhejiang, China. (Supplementary Table 1). The two laboratory strains have been reared on rice seedlings in the laboratory without exposure to any insecticide for more than 10 years^39,40^. All the *N. lugens* strains were reared under a 16 h/8 h light/dark photoperiod at 27 ± 1 °C and 70%–80% relative humidity on Taichung Native 1 (TN1) rice seedlings^41^.

### Insecticide bioassay

Information on the insecticides used in this study is shown in Supplementary Table 2^14^. For insecticide bioassay, the rice‐seeding dip method was performed: insecticides (imidacloprid, nitenpyram, dinotefuran, thiamethoxam, clothianidin, sulfoxaflor, isoprocarb, chlorpyrifos, buprofezin, and etofenprox) or commercial formulation (triflumezopyrim) were dissolved in *N*, *N*‐dimethyl formamide (DMF) or water, respectively. The solutions were then serially diluted with 0.1% Triton X‐100 in water or water only. Fifteen rice seedlings were soaked in different concentrations of insecticide solutions for 30 s, then wrapped with water-impregnated cotton and transferred to plastic cups. Three replicate cups were set up for each concentration and each cup was added 15 third-instar *N. lugens* (F_1_ or F_2_ generation of fields strains). Mortality was recorded after exposure to imidacloprid, nitenpyram, thiamethoxam, clothianidin, dinotefuran, and sulfoxaflor for 96 h, etofenprox, isoprocarb, chlorpyrifos for 72 h, and triflumezopyrim and buprofezin for 120 h. The nymphs were considered dead if they were unable to move after a gentle prodding with a fine brush^14^.

### Sample collection for sequencing

For each of the 11 strains of *N. lugens* (nine field and two laboratory strains), thirty surface-disinfected third–instar nymphs were pooled to provide 3–5 biological replicates for each strain. The collected *N. lugens* FS have been propagated in the laboratory for 1 or 2 generations to generate a sufficient number of insects for the insecticide susceptibility assays, transcriptome sequencing, and microbiome profiling by 16 S sequencing (requiring more than 4000 individuals per strain). To ensure consistency between sequencing results and phenotypic (insecticide susceptibility) data, we subjected insects from the same generation (F_1_ or F_2)_ to the insecticides susceptibility assay and sequencing.

### Transcriptome sequencing

Three micrograms of total RNA were used as input materials for RNA sample preparations. Sequencing libraries were generated using the TruSeq RNA Sample Preparation Kit (Illumina, San Diego, CA, USA). Sequencing was conducted on a Hiseq platform (Illumina) by Shanghai Personal Biotechnology Cp. Ltd.

Clean reads were obtained by removing raw reads with adapters, poly-*N*, and having a low quality (< Q20). Gene expression levels were estimated by the RSEM software package (http://deweylab.biostat.wisc.edu/rsem). Transcripts were annotated based on the reference genome (SAMN13382557), and sequences were annotated to the KEGG ORTHOLOGY (KO) database with the KEGG Automatic Annotation Server.

### Microbiome analysis

Total RNA instead of genomic DNA was extracted using RNAiso Plus (TAKARA, DaLian, China), due to sequencing of total genomic DNA extraction cannot distinguish between live (transcriptionally active) and quiescent or dead microbial (transcriptionally inactive)^6^. cDNA was synthesized with RNA (1 μg) using Hifair™ 1st Strand cDNA Synthesis SuperMix for qPCR (YEASEN, Shanghai, China). Subsequently, PCR amplification of the bacterial 16 S rRNA genes and the fungal ITS1 region was performed using the primers 338 F (5′-ACTCCTACGGGAGGCAGCAG -3′)-806R (5′-GGACTACHVGGGTWTCTAAT-3′) and gITS7 (5′-GTGARTCATCGARTCTTTG-3′)-ITS4 (5′-TCCTCCGCTTATTGATATGC-3′)^42^, respectively. Sample–specific 7-bp barcodes were incorporated into the primers for multiplex sequencing. The PCR products were purified using Vazyme VAHTSTM DNA Clean Beads (Vazyme, Nanjing, China) and quantified using the QuantiT PicoGreen dsDNA Assay Kit (Invitrogen, Carlsbad, CA, USA). Paired-end 2 × 250 bp sequencing was performed using the Illumina NovaSeq platform with NovaSeq 6000 SP Reagent Kit at Shanghai Personal Biotechnology Co., Ltd (Shanghai, China). Bioinformatic analyses were performed using QIIME2 2020.11^43^, according to the official tutorials (https://docs.qiime2.org/2020.11/tutorials/) with slight modifications. Briefly, raw sequence data were demultiplexed using the demux plugin followed by primers cutting with cutadapt plugin^44^. Sequence data were processed with the DADA2 plugin to quality filter, denoised, merged, and chimera removed^45^. Non-singleton amplicon sequence variants (ASVs) were aligned with mafft^46^, and Alpha diversity and beta diversity were analyzed by the diversity plugin with the samples rarefied. Taxonomy was assigned to ASVs using the classify-sklearn naïve Bayes taxonomy classifier in feature-classifier plugin^43^, against the SILVA 138 and Warcup Database for bacteria and fungi, respectively^47^.

### Genetic background analysis

Total genomic DNA was extracted from 20 third-instar individual nymphs using the FastDNA SPIN Kit for soil (MP, Biomedicals, California, USA) following the manufacturer’s protocol. Genetic differences were estimated by Inter-simple Sequence Repeat (ISSR) and the primer sequences were shown in Supplementary Table 3. A binary matrix was built according to the presence or absence of amplified bands with the different primers to calculate the dissimilarity metrics based on Nei Unbiased Genetic Distance by POPGENE (Version 1.31)^48^.

### Statistical analysis

Bioassay data were analyzed using Polo Plus software (version 2.0). The daily average temperatures and precipitation from July to September were obtained from the China Meteorological Administration website (http://data.cma.cn). All distance matrices and principal coordinate analysis (PCoA) based on Bray–Curtis dissimilarity metrics were calculated by the VEGAN package (version 2.5–7). To determine significant differences in β-diversity among different field strains, PERMANOVA was carried out using the “adonis” function of the VEGAN package^49^. Spearman correlation between genes transcripts, LC_50_ of each insecticide (All data were used in correlation analysis between insecticide susceptibility-transcriptome-microbiome, only field strains data were used in environmental abiotic factors correlation analysis because we do not have information on their origins or environment histories) and microbial abundance (bacteria and fungi) was calculated using corAndPvalue^50^ in WGCNA package (version 1.69) and *P* was corrected by Benjamini and Hochberg FDR (BH) method, set threshold as *P*(adj) < 0.05 in subsequent use to remove weak interactions^51^. The network was made in Gephi (version 0.9.2) based on the spearman correlation metrics. All packages were performed in R (version 4.0.2). Data plotting and statistical analyses were performed using GraphPad Prism (version 7.0) and R. Statistical significance is being considered as *P* < 0.05 (*) and *P* < 0.01 (**). The geographical distances among the sampling sites were calculated from the sampling coordinates. Distance-decay relationships were calculated as the linear regression relationships between geographic distance and community similarity^52^. Mantel tests were used to estimate the relative contributions of genetic background to microbiome composition based on Bray–Curtis dissimilarity metrics by the Pearson method with 999 permutations.

### Reporting summary

Further information on research design is available in the Nature Research Reporting Summary linked to this article.

## Supplementary information

Reporting Summary

Supplementary Information

Supplementary Data

Supplementary Data Caption 1-5

## Data Availability

All code used for the generation of the study’s analyses is available at https://github.com/pesticidescience/MTNL.

## Data availability

Raw sequence data reported in this paper have been deposited (PRJCA006986) in the Genome Sequence Archive in the BIG Data Center, Chinese Academy of Sciences, under accession codes CRA005260 (16S rRNA and ITS gene sequencing) and CRA005259 (transcriptome sequencing) that are publicly accessible at http://bigd.big.ac.cn/gsa.
