# Supplementary material for: Microbiome variation correlates with the insecticide susceptibility in different geographic strains of a significant agricultural pest, Nilaparvata lugens
Source: NPJ Biofilms Microbiomes. 2023 Jan 12;9:2. doi: 10.1038/s41522-023-00369-5 (PMC9837087; doi:10.1038/s41522-023-00369-5)
Supplement: Supplementary file 1 — Reporting Summary [file 41522_2023_369_MOESM1_ESM.pdf]

nature portfolio

Corresponding author(s): Hu Wan

Last updated by author(s): Dec 10, 2022

Reporting Summary

Nature Portfolio wishes to improve the reproducibility of the work that we publish. This form provides structure for consistency and transparency in reporting. For further information on Nature Portfolio policies, see our Editorial Policies and the Editorial Policy Checklist.

Statistics

For all statistical analyses, confirm that the following items are present in the figure legend, table legend, main text, or Methods section.

n/a	Confirmed	
☐	☒	The exact sample size (*n*) for each experimental group/condition, given as a discrete number and unit of measurement
☐	☒	A statement on whether measurements were taken from distinct samples or whether the same sample was measured repeatedly
☐	☒	The statistical test(s) used AND whether they are one- or two-sided<br>*Only common tests should be described solely by name; describe more complex techniques in the Methods section.*
☐	☒	A description of all covariates tested
☐	☒	A description of any assumptions or corrections, such as tests of normality and adjustment for multiple comparisons
☐	☒	A full description of the statistical parameters including central tendency (e.g. means) or other basic estimates (e.g. regression coefficient) AND variation (e.g. standard deviation) or associated estimates of uncertainty (e.g. confidence intervals)
☐	☒	For null hypothesis testing, the test statistic (e.g. *F*, *t*, *r*) with confidence intervals, effect sizes, degrees of freedom and *P* value noted<br>*Give P values as exact values whenever suitable.*
☒	☐	For Bayesian analysis, information on the choice of priors and Markov chain Monte Carlo settings
☒	☐	For hierarchical and complex designs, identification of the appropriate level for tests and full reporting of outcomes
☐	☒	Estimates of effect sizes (e.g. Cohen's *d*, Pearson's *r*), indicating how they were calculated

Our web collection on statistics for biologists contains articles on many of the points above.

Software and code

Policy information about availability of computer code

Data collection	Transcriptome sequencing was conducted on a Hiseq platform (Illumina) by Shanghai Personal Biotechnology Cp. Ltd.<br>Amplification sequencing of bacterial 16S rRNA genes and the fungal ITS1 region was performed using the Illlumina NovaSeq platform with NovaSeq 6000 SP Reagent Kit at Shanghai Personal Biotechnology Co., Ltd (Shanghai, China).
Data analysis	Gene expression levels were estimated by RSEM software package (http://deweylab. biostat.wisc.edu/rsem). Transcripts were annotated based on the reference genome (SAMN13382557), and sequences were annotated to the KEGG ORTHOLOGY (KO) database with the KEGG Automatic Annotation Server.<br>Bioinformatic analyses of microbiome were performed using QIIME2 2020.1143, according to the official tutorials (https://docs.qiime2.org/2020.11/tutorials/).<br>Genetic background analysis were carried out in POPGENE (Version 1.31).<br>Bio-assay data were analyzed using Polo Plus software (version 2.0).<br>Most data plotting and statistical analyses were performed using GraphPad Prism (version 7.0).<br>All other packages on data analyses were performed in R (version 4.0.2).

For manuscripts utilizing custom algorithms or software that are central to the research but not yet described in published literature, software must be made available to editors and reviewers. We strongly encourage code deposition in a community repository (e.g. GitHub). See the Nature Portfolio guidelines for submitting code & software for further information.

Data

Policy information about availability of data

All manuscripts must include a data availability statement. This statement should provide the following information, where applicable:

- Accession codes, unique identifiers, or web links for publicly available datasets
- A description of any restrictions on data availability
- For clinical datasets or third party data, please ensure that the statement adheres to our policy

Raw sequence data reported in this paper have been deposited (PRJCA006986) in the Genome Sequence Archive in the BIG Data Center, Chinese Academy of Sciences under accession codes CRA005260 (16S rRNA and ITS gene sequencing) and CRA005259 (transcriptome sequencing) that are publicly accessible at http://bigd.big.ac.cn/gsa.

Field-specific reporting

Please select the one below that is the best fit for your research. If you are not sure, read the appropriate sections before making your selection.

☒ Life sciences ☐ Behavioural & social sciences ☐ Ecological, evolutionary & environmental sciences

For a reference copy of the document with all sections, see nature.com/documents/nr-reporting-summary-flat.pdf

Life sciences study design

All studies must disclose on these points even when the disclosure is negative.

Sample size	For each of the 11 strains of N. lugens (nine field and two laboratory strains), thirty surface-disinfected third–instar nymphs were pooled to provide 3–5 biological replicates for each strain. The collected N. lugens field strains have been propagated in the laboratory for 1 or 2 generations to generate sufficient number of insects for the insecticide susceptibility assays, transcriptome sequencing and microbiome profiling by 16S sequencing (requiring more than 4,000 individuals per strain).
Data exclusions	No data were excluded from the analyses.
Replication	All sequencing data and R code used for data analyses are available Genome Sequence Archive in the BIG Data Center, Chinese Academy of Sciences (PRJCA006986) and GitHub (https://github.com/pesticidescience/MTNL.), respectively. All attempts at replication were successful.
Randomization	N. lugens in all studies were randomized selected in a specific age group.
Blinding	All authors approved group allocation during data collection and analysis.

Reporting for specific materials, systems and methods

We require information from authors about some types of materials, experimental systems and methods used in many studies. Here, indicate whether each material, system or method listed is relevant to your study. If you are not sure if a list item applies to your research, read the appropriate section before selecting a response.

Materials & experimental systems

n/a	Involved in the study	
☒	☐	Antibodies
☒	☐	Eukaryotic cell lines
☒	☐	Palaeontology and archaeology
☐	☒	Animals and other organisms
☒	☐	Human research participants
☒	☐	Clinical data
☒	☐	Dual use research of concern

Methods

n/a	Involved in the study	
☒	☐	ChIP-seq
☒	☐	Flow cytometry
☒	☐	MRI-based neuroimaging

Animals and other organisms

Policy information about studies involving animals; ARRIVE guidelines recommended for reporting animal research

Laboratory animals	The laboratory strain 1 (LS1) and laboratory strain 2 (LS2) of N. lugens.
Wild animals	The study did not involve wild animals.

Field-collected samples	N. lugens field strains were collected from rice paddy fields from nine locations in six provinces in China in 2019.
Ethics oversight	No ethical approval or guidance was required. The did not involved any legally protected insect species.

Note that full information on the approval of the study protocol must also be provided in the manuscript.
