# Supplementary material for: Microbiome variation correlates with the insecticide susceptibility in different geographic strains of a significant agricultural pest, Nilaparvata lugens
Source: NPJ Biofilms Microbiomes. 2023 Jan 12;9:2. doi: 10.1038/s41522-023-00369-5 (PMC9837087; doi:10.1038/s41522-023-00369-5)
Supplement: Supplementary file 2 — Supplementary Information [file 41522_2023_369_MOESM2_ESM.pdf]

Supplementary Information files for:

Microbiome variation correlates with the insecticide susceptibility in different geographic strains of a significant agricultural pest, *Nilaparvata lugens*

Yunhua Zhang [1, 2, #], Tingwei Cai [1, 2, #], Maojun Yuan [1, 2], Zhao Li [1, 2], Ruoheng Jin [1, 2], Zhijie Ren [1, 2], Yao Qin [1, 2], Chang Yu [1, 2], Yongfeng Cai [1, 2], Runhang Shu [3], Shun He [2], Jianhong Li [2], Adam C.N. Wong [3], Hu Wan [1, 2 *]

[1] *State Key Laboratory of Agricultural Microbiology, Huazhong Agricultural University, Wuhan 430070, China*

[2] *Hubei Insect Resources Utilization and Sustainable Pest Management Key Laboratory, College of Plant Science and Technology, Huazhong Agricultural University, Wuhan 430070, China*

[3] *Department of Entomology and Nematology, University of Florida, Gainesville, Florida 32611, United States*

* Corresponding author.

E–mail: huwan@mail.hzau.edu.cn (H. Wan)

[#] These authors contributed equally to this paper.

Supplementary Table1 Information of sample collection

Sample	Province	City	Average daily precipitation (mm)	Average daily temperature (℃)	Latitude	Longitude	Data (Year/Month/Day)
HBXG	Hubei	Xiaogan	1.03	28.78	29.57° N	115.36° E	2019/8/10
HBCB	Hubei	Chibi	1.26	27.65	31.31° N	113.81° E	2019/9/21
HBYX	Hubei	Yangxin	1.17	28.64	29.48° N	113.55° E	2019/9/24
HBCY	Hubei	Chongyang	1.03	28.78	29.85° N	115.20° E	2019/7/6
HNCS	Hunan	Changsha	0.94	28.78	29.54° N	114.04° E	2019/8/26
JXNC	Jiangxi	Nanchang	3.16	28.48	28.32° N	115.58° E	2019/9/17
GDSG	Guangdong	Shaoguan	3.80	26.18	24.80° N	113.80° E	2019/8/30
GZHP	Guizhou	Huangping	2.64	22.89	27.90° N	107.90° E	2019/7/10
HNXY	Henan	Xinyang	1.31	27.02	31.58° N	115.24° E	2019/9/7
LS1 (Lab strain 1)	Hubei	Wuhan	\	28.00	\	\	2008
LS2 (Lab strain 2)	Zhejiang	Hangzhou	\	28.00	\	\	1995

Supplementary Table2 Insecticides information used in this study

Insecticide group	Insecticide	Classification	Target	Supplier	Address	Content (%)
Neonicotinoids	Imidacloprid	4A	Nicotinic acetylcholine receptor (nAChR)	Hubei Kangbaotai Fine-Chemicals Co., Ltd.	Wuhan, China	95.8
	Nitenpyram	4A				95.8
	Dinotefuran	4A				91
	Thiamethoxam	4A				95
	Clothianidin	4A				96
Sulfoximines	Sulfoxaflor	4C				96
Mesoionics	Triflumezopyrim	4E		Du Pont Co., Ltd.	Shanghai, China	10, aqueous suspension concentrate
Carbamates	Isoprocarb	1A	Acetylcholinesterase (AChE)	Hubei Kangbaotai Fine-Chemicals Co., Ltd.	Wuhan, China	97.3
Organophosphates	Chlorpyrifos	1B				98
Insect growth regulator	Buprofezin	16	Chitin biosynthesis			97.4
Pyrethroids	Etofenprox	3A	Sodium channel			95

Supplementary Table3 Primers for Inter-simple Sequence Repeat (ISSR)

Primer name	Primer sequences (5’-3’)
ISSR U825	ACACACACACACACACT
ISSR U859	TGTGTGTGTGTGTGTGRC
ISSR U861	ACCACCACCACCACCACC
ISSR U846	CACACACACACACACART

Supplementary Table4 Susceptibility of *Nilaparvata lugens* to imidacloprid

Strain	N	LC_{50} (95% CI) mg/L	Slope (SE)	χ^2 (df)
HBCB	270	295.91 (236.47-390.15)	1.95 (0.25)	5.33 (3)
GZHP	270	207.67 (171.02-261.63)	2.59 (0.34)	5.50 (3)
HBCY	270	249.85 (150.05-661.59)	2.03 (0.26)	16.70 (3)
HNCS	270	500.50 (240.61-614.01)	2.61 (0.30)	6.48 (3)
JXNC	270	395.35 (324.26-484.86)	2.30 (0.29)	4.11 (3)
GDSG	270	287.90 (226.16-353.32)	2.25 (0.29)	10.75 (3)
HBYX	270	529.51 (421.75-707.48)	2.40 (0.35)	3.49 (3)
HNXY	270	447.04 (324.59-747.47)	1.98 (0.33)	1.48 (3)
HBXG	270	266.99 (194.46-402.87)	1.57 (0.24)	5.67 (2)
LS2	270	25.01 (19.90-29.92)	2.91 (0.33)	7.63 (3)
LS1	270	42.91 (32.49-52.68)	2.67 (0.32)	3.12 (3)

N: Number of insects in bioassay

Supplementary Table5 Susceptibility of *Nilaparvata lugens* to thiamethoxam

Strain	N	LC_{50} (95% CI) mg/L	Slope (SE)	χ2 (df)
HBCB	270	52.73 (36.32-87.05)	1.14 (0.25)	7.45 (3)
GZHP	270	116.42 (95.65-141.16)	2.62 (0.32)	2.35 (3)
HBCY	270	49.66 (38.04-58.02)	6.00 (0.99)	0.06 (2)
HNCS	270	80.20 (52.92-183.99)	1.82 (0.44)	2.33 (3)
JXNC	270	222.61 (171.04-359.11)	2.74 (0.52)	1.88 (2)
GDSG	270	116.69 (90.34-161.85)	2.11 (0.32)	5.16 (3)
HBYX	270	119.42 (90.36-170.93)	1.81 (0.28)	11.32 (3)
HNXY	270	29.04 (22.88-35.53)	2.20 (0.26)	0.50 (3)
HBXG	270	27.85 (16.48-40.57)	1.65 (0.35)	2.31 (3)
LS2	270	2.41 (1.87-2.92)	2.57 (0.30)	1.11 (3)
LS1	270	4.45 (3.33-5.52)	2.33 (0.29)	0.15 (3)

N: Number of insects in bioassay

Supplementary Table6 Susceptibility of *Nilaparvata lugens* to nitenpyram

Strain	N	LC_{50} (95% CI) mg/L	Slope (SE)	χ^2 (df)
HBCB	270	4.87 (3.77-5.95)	2.44 (0.29)	2.86 (3)
GZHP	270	8.30 (5.93-14.16)	1.46 (0.26)	4.83 (3)
HBCY	270	6.36 (5.11-8.40)	2.08 (0.26)	0.86 (3)
HNCS	270	6.50 (5.17-7.97)	2.13 (0.26)	3.80 (3)
JXNC	270	2.63 (2.04-3.27)	0.80 (0.25)	9.11 (3)
GDSG	270	6.89 (5.29-8.75)	2.27 (0.32)	2.22 (3)
HBYX	270	10.05 (7.50-14.21)	1.74 (0.29)	1.70 (3)
HNXY	270	6.27 (4.96-8.12)	2.55 (1.00)	0.35 (2)
HBXG	270	5.95 (4.78-7.21)	2.58 (0.30)	1.52 (3)
LS2	270	0.57 (0.46-0.69)	2.48 (0.29)	8.51 (3)
LS1	270	0.98 (0.84-1.16)	3.40 (0.37)	1.61 (3)

N: Number of insects in bioassay

Supplementary Table7 Susceptibility of *Nilaparvata lugens* to sulfoxaflor

Strain	N	LC_{50} (95% CI) mg/L	Slope (SE)	χ^2 (df)
HBCB	270	7.97 (4.51-12.92)	3.61 (0.43)	14.60 (2)
GZHP	270	7.76 (6.38-9.41)	2.62 (0.32)	2.35 (3)
HBCY	270	7.42 (6.29-8.94)	2.89 (0.32)	8.31 (3)
HNCS	270	9.31 (8.15-10.80)	4.31 (0.50)	10.76 (3)
JXNC	270	15.49 (12.21-21.96)	2.35 (0.33)	1.65 (3)
GDSG	270	14.68 (10.68-23.61)	1.72 (0.30)	3.53 (3)
HBYX	270	10.14 (8.41-12.53)	1.65 (0.28)	2.49 (3)
HNXY	270	9.13 (7.11-13.37)	2.54 (0.45)	0.80 (2)
HBXG	270	6.98 (5.91-8.15)	3.24 (0.35)	0.93 (3)
LS2	270	2.61 (2.06-3.17)	2.48 (0.29)	1.69 (3)
LS1	270	3.06 (2.46-3.70)	2.38 (0.27)	3.43 (3)

N: Number of insects in bioassay

Supplementary Table8 Susceptibility of *Nilaparvata lugens* to dinotefuran

Strain	N	LC_{50} (95% CI) mg/L	Slope (SE)	χ^2 (df)
HBCB	270	19.45 (15.54-24.25)	1.97 (0.24)	0.12 (3)
GZHP	270	14.96 (12.21-18.91)	2.38 (0.31)	1.52 (3)
HBCY	270	11.22 (8.56-13.74)	2.58 (0.31)	5.08 (3)
HNCS	270	35.21 (27.52-44.26)	1.85 (0.24)	3.97 (3)
JXNC	270	20.17 (15.67-25.99)	1.92 (0.28)	2.40 (3)
GDSG	270	29.71 (23.96-39.23)	2.78 (0.38)	2.95 (3)
HBYX	270	41.87 (30.19-70.19)	1.80 (0.31)	3.13 (3)
HNXY	270	29.00 (21.36-31.88)	1.39 (0.18)	3.32 (3)
HBXG	270	34.72 (20.32-66.60)	0.93 (0.20)	3.98 (3)
LS2	270	1.17 (0.89-1.46)	2.24 (0.27)	1.91 (3)
LS1	270	2.47 (1.94-2.98)	2.66 (0.31)	3.17 (3)

N: Number of insects in bioassay

Supplementary Table9 Susceptibility of *Nilaparvata lugens* to clothianidin

Strain	N	LC_{50} (95% CI) mg/L	Slope (SE)	χ^2 (df)
HBCB	270	15.59 (12.83-18.49)	2.78 (0.31)	4.15 (3)
GZHP	270	26.60 (20.30-33.96)	2.31 (0.29)	4.01 (3)
HBCY	270	169.23 (88.53-520.39)	0.67 (0.22)	4.39 (3)
HNCS	270	132.00 (107.24-172.40)	2.31 (0.28)	2.90 (3)
JXNC	270	66.42 (51.79-92.75)	1.88 (0.27)	1.22 (3)
GDSG	270	32.27 (23.31-42.66)	1.72 (0.30)	3.53 (3)
HBYX	270	45.74 (36.47-58.46)	2.39 (0.32)	1.05 (3)
HNXY	270	26.10 (20.24-32.01)	2.26 (0.27)	1.54 (3)
HBXG	270	18.32 (11.91-24.07)	2.32 (0.46)	0.22 (2)
LS2	270	0.94 (0.77-1.15)	2.38 (0.27)	5.64 (3)
LS1	270	4.31 (3.18-5.41)	2.21 (0.28)	5.53 (3)

N: Number of insects in bioassay

Supplementary Table10 Susceptibility of *Nilaparvata lugens* to etofenprox

Strain	N	LC_{50} (95% CI) mg/L	Slope (SE)	χ^2 (df)
HBCB	270	39.32 (17.68-59.95)	2.84 (0.51)	2.75 (3)
GZHP	270	85.33 (66.90-109.96)	1.88 (0.26)	2.99 (3)
HBCY	270	50.30 (25.82-74.10)	1.50 (0.25)	6.62 (3)
HNCS	270	65.53 (48.57-83.12)	1.82 (0.24)	7.95 (3)
JXNC	270	105.31 (86.77-128.65)	2.60 (0.32)	4.26 (3)
GDSG	270	69.54 (55.20-86.00)	2.20 (0.28)	10.88 (3)
HBYX	270	65.25 (48.13-82.55)	1.88 (0.21)	1.49 (4)
HNXY	270	128.39 (102.94-152.93)	2.98 (0.34)	0.88 (3)
HBXG	270	76.73 (42.63-108.09)	2.10 (0.36)	1.31 (3)
LS2	270	21.11 (16.28-25.97)	2.30 (0.34)	0.60 (3)
LS1	270	104.47 (78.73-128.30)	2.76 (0.33)	1.14 (3)

N: Number of insects in bioassay

Supplementary Table11 Susceptibility of *Nilaparvata lugens* to isoprocorb

Strain	N	LC_{50} (95% CI) mg/L	Slope (SE)	χ^2 (df)
HBCB	270	111.50 (61.82-157.45)	2.29 (0.35)	1.48 (3)
GZHP	270	242.56 (199.96-302.03)	2.57 (0.33)	0.30 (3)
HBCY	270	258.93 (202.06-316.98)	2.20 (0.27)	10.72 (3)
HNCS	270	642.64 (531.82-821.60)	2.78 (0.34)	1.70 (3)
JXNC	270	254.87 (203.69-331.02)	2.07 (0.28)	1.99 (3)
GDSG	270	225.44 (160.88-287.90)	2.39 (0.35)	1.85 (3)
HBYX	270	188.84 (119.19-254.51)	2.00 (0.33)	5.67 (3)
HNXY	270	236.00 (162.37-308.86)	1.91 (0.31)	3.56 (3)
HBXG	270	324.99 (265.50-389.84)	3.34 (0.43)	2.07 (3)
LS2	270	20.67 (15.19-25.70)	2.49 (0.37)	6.46 (2)
LS1	270	117.58 (92.59-140.92)	3.06 (0.36)	0.58 (3)

N: Number of insects in bioassay

Supplementary Table12 Susceptibility of *Nilaparvata lugens* to chlorpyrifos

Strain	N	LC_{50} (95% CI) mg/L	Slope (SE)	χ^2 (df)
HBCB	270	19.90 (14.81-24.54)	2.85 (0.35)	2.15 (3)
GZHP	270	18.82 (15.56-22.60)	2.82 (0.35)	3.52 (3)
HBCY	270	29.33 (24.19-34.50)	3.10 (0.34)	1.90 (3)
HNCS	270	41.81 (28.38-62.87)	2.54 (0.28)	11.83 (3)
JXNC	270	21.45 (16.13-26.24)	3.14 (0.40)	1.68 (3)
GDSG	270	30.21 (21.10-36.90)	5.89 (0.97)	0.24 (3)
HBYX	270	24.76 (12.00-37.03)	2.36 (0.44)	7.12 (2)
HNXY	270	42.36 (31.20-68.28)	2.03 (0.33)	2.58 (3)
HBXG	270	13.58 (10.84-16.34)	2.59 (0.30)	5.94 (3)
LS2	270	7.35 (6.45-8.74)	3.50 (0.38)	7.50 (3)

LS1	270	25.01 (19.90-29.92)	2.92 (0.33)	7.63 (3)

N: Number of insects in bioassay

Supplementary Table13 Susceptibility of *Nilaparvata lugens* to buprofezin

Strain	N	LC_{50} (95% CI) mg/L	Slope (SE)	χ^2 (df)
HBCB	270	89.00 (46.55-142.26)	0.86 (0.24)	2.00 (3)
GZHP	270	111.85 (74.16-196.57)	1.01 (0.22)	5.89 (3)
HBCY	270	231.33 (166.78-424.43)	2.35 (0.42)	0.31 (2)
HNCS	270	78.22 (38.21-123.94)	0.90 (0.22)	3.80 (3)
JXNC	270	76.46 (57.83-122.11)	2.02 (0.38)	0.99 (2)
GDSG	270	84.04 (54.37-117.25)	1.25 (0.23)	0.94 (3)
HBYX	270	25.80 (17.49-33.46)	1.87 (0.32)	2.83 (2)
HNXY	270	79.00 (62.68-103.53)	1.81 (0.24)	2.85 (3)
HBXG	270	48.35 (33.37-66.35)	1.26 (0.21)	2.23 (3)
LS2	270	5.11 (3.87-6.38)	2.03 (0.26)	1.29 (3)
LS1	270	15.69 (12.80-18.78)	2.57 (0.29)	1.49 (4)

N: Number of insects in bioassay

Supplementary Table14 Susceptibility of *Nilaparvata lugens* to triflumezopyrim

Strain	N	LC_{50} (95% CI) mg/L	Slope (SE)	χ^2 (df)
HBCB	270	0.12 (0.08-0.16)	1.56 (0.24)	2.00 (3)
GZHP	270	0.10 (0.08-0.12)	2.49 (0.31)	8.31 (3)
HBCY	270	0.14 (0.11-0.18)	2.07 (0.25)	1.53 (3)
HNCS	270	0.20 (0.15-0.29)	1.88 (0.26)	0.76 (3)
JXNC	270	0.11 (0.08-0.14)	1.76 (0.26)	4.86 (3)
GDSG	270	0.07 (0.06-0.09)	2.50 (0.31)	2.81 (3)
HBYX	270	0.14 (0.11-0.17)	2.22 (0.26)	1.14 (3)
HNXY	270	0.11 (0.09-0.13)	2.36 (0.27)	0.72 (3)
HBXG	270	0.12 (0.09-0.16)	2.01 (0.30)	3.61 (3)
LS2	270	0.060 (0.046-0.073)	2.35 (0.28)	2.37 (3)
LS1	270	0.049 (0.033-0.065)	1.70 (0.25)	4.08 (3)

N: Number of insects in bioassay

Supplementary Table15 Sample information of transcriptome sequencing

Sample	Reads No.	Bases (bp)	Q30 (bp)	N (%)	Q20 (%)	Q30 (%)
HBCB3	539,435,16	809,152,740,0	758,079,980,3	0.00061	97.48	93.68
HBCB4	459,680,20	689,520,300,0	645,652,458,8	0.00059	97.32	93.63
HBCB5	527,087,02	790,630,530,0	742,513,089,2	0.00061	97.50	93.91
GZHP1	459,577,92	689,366,880,0	648,966,768,5	0.00061	97.65	94.13
GZHP2	538,365,76	807,548,640,0	757,263,647,0	0.00061	97.53	93.77
GZHP5	497,486,08	746,229,120,0	701,516,155,0	0.00061	97.57	94.00

HBCY2	446,631,44	669,947,160,0	627,115,844,1	0.0011	97.50	93.60
HBCY3	459,393,70	689,090,550,0	635,136,578,5	0.0011	96.99	92.17
HBCY5	490,103,24	735,154,860,0	685,808,179,8	0.0011	97.32	93.28
HNCS2	521,480,98	782,221,470,0	734,557,986,8	0.00061	97.55	93.90
HNCS4	540,566,20	810,849,300,0	756,380,197,0	0.00061	97.25	93.28
HNCS5	465,529,98	698,294,970,0	657,201,300,6	0.00060	97.57	94.11
JXNC1	438,824,52	658,236,780,0	605,668,962,0	0.00060	96.68	92.01
JXNC4	489,341,92	734,012,880,0	688,739,351,9	0.00061	97.53	93.83
JXNC5	431,195,94	646,793,910,0	605,706,226,2	0.00061	97.38	93.64
GDSG1	453,411,16	680,116,740,0	640,112,082,2	0.00060	97.59	94.11
GDSG3	564,350,08	846,525,120,0	795,414,748,0	0.00061	97.54	93.96
GDSG4	455,388,78	683,083,170,0	643,830,829,7	0.00059	97.65	94.25
HBYX1	432,170,84	648,256,260,0	606,715,767,5	0.0013	97.42	93.59
HBYX3	457,591,84	686,387,760,0	641,098,735,3	0.0011	97.34	93.40
HBYX5	508,990,92	763,486,380,0	714,524,174,3	0.0011	97.46	93.58
HNXY1	555,840,46	833,760,690,0	779,533,957,6	0.00061	97.37	93.49
HNXY2	549,229,24	823,843,860,0	769,017,298,7	0.00061	97.26	93.34
HNXY3	525,124,00	787,686,000,0	739,573,801,9	0.00061	97.51	93.89
HBXG1	426,479,20	639,718,800,0	600,342,328,0	0.0013	97.65	93.84
HBXG3	483,867,62	725,801,430,0	681,035,168,3	0.0011	97.70	93.83
HBXG4	501,102,68	751,654,020,0	704,738,372,7	0.0011	97.62	93.75
LS22	568,929,08	853,393,620,0	801,804,301,4	0.00061	97.64	93.95
LS24	517,178,98	775,768,470,0	728,026,770,5	0.00061	97.57	93.84
LS25	519,578,52	779,367,780,0	728,178,180,5	0.00061	97.38	93.43
LS11	470,766,24	706,149,360,0	667,567,734,6	0.00059	97.86	94.53
LS12	501,283,72	751,925,580,0	707,794,346,0	0.00062	97.65	94.13
LS13	519,454,52	779,181,780,0	728,592,186,7	0.00061	97.36	93.50

Reads No.: Total reads number; Bases (bp): Total bases number; Q30 (bp): Base recognition accuracy is more than 99.9% of the total number of bases; N (%): Percentage of fuzzy base; Q20 (%): Percentage of base recognition accuracy is more than 99% of the total number of bases; Q30 (%): Percentage of base recognition accuracy is more than 99.9% of the total number of bases.

Supplementary Table16 α diversity of microbiome in *Nilaparvata lugens*

		Fungi					Bacteria		
Sample	Shannon	Faith pd	Pielou evenness	Observed ASVs	Sample	Shannon	Faith pd	Pielou evenness	Observed ASVs
HBCB1	1.97	15.92	0.32	73.00	HBCB1	3.92	10.90	0.58	104.00
HBCB2	3.18	11.51	0.57	47.00	HBCB2	4.08	11.28	0.58	127.00
HBCB3	3.50	9.24	0.73	28.00	HBCB3	3.44	9.69	0.52	102.00
HBCB4	2.94	15.64	0.49	62.00	HBCB4	3.25	10.59	0.49	99.00
HBCB5	1.04	8.56	0.19	47.00	HBCB5	4.77	12.22	0.66	144.00
GZHP1	2.01	9.21	0.35	53.00	GZHP1	0.48	4.50	0.12	15.00
GZHP2	3.30	12.58	0.58	51.00	GZHP2	0.65	4.81	0.14	27.00
GZHP3	1.49	13.42	0.26	51.00	GZHP3	0.77	4.70	0.17	23.00
GZHP4	2.43	8.49	0.42	54.00	GZHP4	0.58	4.44	0.15	16.00
GZHP5	2.61	8.37	0.46	49.00	GZHP5	0.73	4.99	0.15	27.00
HBCY2	3.84	9.72	0.73	38.00	HBCY2	1.12	5.01	0.25	23.00
HBCY3	2.25	9.96	0.39	55.00	HBCY3	0.96	4.83	0.21	24.00
HBCY4	2.07	9.92	0.36	51.00	HBCY4	0.85	5.58	0.18	27.00
HBCY5	2.05	9.46	0.38	43.00	HBCY5	1.10	5.80	0.22	30.00
HNCS2	2.49	14.15	0.40	76.00	HNCS2	0.97	4.43	0.24	17.00
HNCS3	2.94	14.86	0.47	76.00	HNCS3	1.55	6.14	0.30	38.00
HNCS4	0.78	18.03	0.12	85.00	HNCS4	2.32	5.49	0.46	33.00
JXNC1	2.01	12.27	0.33	68.00	JXNC1	1.04	6.21	0.19	45.00
JXNC2	0.74	9.54	0.13	47.00	JXNC2	3.15	8.60	0.49	86.00
JXNC3	3.58	18.33	0.62	56.00	JXNC3	3.00	7.46	0.49	70.00
JXNC4	1.10	12.32	0.18	76.00	JXNC4	0.75	5.00	0.17	22.00
JXNC5	1.42	7.92	0.23	69.00	JXNC5	3.86	9.78	0.58	102.00
GDSG1	1.29	7.18	0.27	27.00	GDSG1	2.13	6.76	0.38	47.00
GDSG2	1.14	8.35	0.21	40.00	GDSG2	1.57	5.60	0.33	28.00
GDSG3	1.15	11.01	0.21	49.00	GDSG3	1.82	6.08	0.35	39.00
GDSG4	2.14	13.17	0.36	59.00	GDSG4	1.72	5.96	0.34	35.00
HBYX1	1.75	14.00	0.30	54.00	HBYX1	1.59	5.09	0.31	34.00
HBYX2	1.14	8.63	0.20	55.00	HBYX2	0.60	5.02	0.13	24.00
HBYX3	0.96	7.13	0.17	47.00	HBYX3	1.43	5.51	0.29	32.00
HBYX4	0.68	9.38	0.12	54.00	HBYX4	1.69	5.93	0.34	32.00
HBYX5	1.24	10.33	0.21	64.00	HBYX5	1.39	4.58	0.31	23.00
HNXY1	1.63	9.03	0.27	65.00	HNXY1	0.54	5.47	0.11	27.00
HNXY2	1.88	11.70	0.30	72.00	HNXY2	0.54	5.18	0.12	25.00
HNXY3	1.80	9.47	0.31	53.00	HNXY3	0.45	4.63	0.11	18.00
HNXY4	1.51	11.19	0.24	73.00	HNXY4	0.96	6.43	0.18	38.00
HNXY5	1.85	11.57	0.32	55.00	HNXY5	0.52	5.34	0.11	23.00
HBXG1	1.70	14.28	0.28	70.00	HBXG1	1.03	4.41	0.24	21.00
HBXG2	1.40	9.30	0.24	57.00	HBXG2	1.63	5.09	0.36	22.00
HBXG3	3.09	12.07	0.51	64.00	HBXG3	1.55	5.08	0.32	28.00
HBXG4	0.95	12.26	0.16	61.00	HBXG4	1.33	5.21	0.30	21.00

HBXG5	1.70	13.63	0.27	80.00	HBXG5	1.66	5.09	0.34	28.00
LS21	3.21	16.22	0.57	48.00	LS21	5.49	10.56	0.75	159.00
LS22	2.88	16.40	0.48	65.00	LS22	4.07	9.89	0.59	116.00
LS23	4.03	15.03	0.67	65.00	LS23	4.12	9.56	0.61	112.00
LS24	2.76	18.04	0.42	100.00	LS24	3.78	9.37	0.56	110.00
LS25	2.54	16.78	0.39	92.00	LS25	0.71	5.35	0.16	22.00
LS11	3.16	16.63	0.51	75.00	LS11	1.23	5.99	0.24	34.00
LS12	3.33	11.31	0.58	55.00	LS12	1.09	5.81	0.22	33.00
LS13	4.17	13.99	0.71	60.00	LS13	1.49	6.54	0.28	40.00
LS14	3.06	15.79	0.50	68.00	LS14	1.42	6.03	0.27	36.00
LS15	3.40	13.06	0.58	57.00	LS15	1.25	6.26	0.24	36.00

Supplementary Table17 Core microbiome of *Nilaparvata lugens*

**Fungi**	**Bacteria**
g__*Hirsutella*	g__*Rhodococcus*
g__*Hypocrea*	g__*Chitinophaga*
g__*Aspergillus*	s__*Siphonobacter_aquaeclarae*
g__*Cladosporium*	s__*cf._Chryseobacterium*
g__*Frantisekia*	g__*Chryseobacterium*
g__*Davidiella*	g__*Sphingobacterium*
g__*Penicillium*	s__env.OPS_17
g__*Raffaelea*	g__*Chloroplast*
g__*Gibberella*	g__*Staphylococcus*
g__*Cryptococcus*	s__*Staphylococcus_sciuri*
g__*Lecanicillium*	g__*Allorhizobium.Neorhizobium.Pararhizobium.Rhizobium*
	s__*proteobacterium_symbiont*
	g__*Sphingomonas*
	s__*Zavarzinia_compransoris*
	g__*Rivicola*
	g__*Burkholderia.Caballeronia.Paraburkholderia*
	g__*Cupriavidus*
	f__Comamonadaceae
	g__*Acidovorax*
	g__*Delftia*
	s__*Pseudacidovorax_intermedius*
	g__*Methylophilus*
	g__*Herbaspirillum*
	f__Enterobacteriaceae
	g__*Escherichia.Shigella*
	g__*Pantoea*
	g__*Arsenophonus*
	g__*Serratia*
	g__*Acinetobacter*

s__*Acinetobacter_baylyi*
s__*Acinetobacter_soli*
g__*Pseudomonas*
s__*Pseudomonas_otitidis*
g__*Hydrocarboniphaga*

Supplementary Table18 Detoxifying genes involved in insecticide resistance of *Nilaparvata lugens*

**Insecticides**	**Detoxifying genes**
Etofenprox	*CYP6FU1, CYP425A1* and *CYP6AY1* [1]
Sulfoxaflor	*CYP6ER1* [2]
Nitenpyram	*CYP6ER1* [3]
Clothianidin	*CYP6ER1* [4]
Chlorpyrifos	*NlCarE* [5]
Imidacloprid	*CYP6AY1, CYP6ER1, CYP4CE1* and *CYP6CW1* [6]
Pyrethroid	*nlgst1-1* [7]
Nitenpyram	*NlCarE1* and *NlCarE19* [8]
Beta-Cypermethrin and Imidacloprid	*CPR* [9]

Supplementary Table19 The effect of genetic background on core microbiome of *Nilaparvata lugens* to insecticides via Mantel test analysis.

**Effect**	**Variation**	**r**	***P***
Bacteria	Genetic background	0.26	0.051
Fungi	Genetic background	0.26	0.053

Reference

1. Sun, H., Yang, B., Zhang, Y. & Liu, Z. Metabolic resistance in *Nilaparvata lugens* to etofenprox, a non-ester pyrethroid insecticide. *Pestic. Biochem. Physiol.* **136**, 23–28 (2016).

2. Liao, X., Jin, R., Zhang, X., Ali, E. & Wan, H. Characterization of sulfoxaflor resistance in the brown planthopper, *Nilaparvata lugens* (stål). *Pest Manag. Sci.* **75**, 1646–1654 (2018).

3. Mao, K. *et al.* Characterization of nitenpyram resistance in *Nilaparvata lugens* (stål).

Pestic. Biochem. Physiol. **157**, 23–26 (2019).

4. Jin, R. *et al.* Overexpression of *CYP6ER1* associated with clothianidin resistance in *Nilaparvata lugens* (Stål). *Pestic. Biochem. Physiol.* **154**, 39–45 (2019).
5. Lu, K. *et al.* Characterization and functional analysis of a carboxylesterase gene associated with chlorpyrifos resistance in *Nilaparvata lugens* (Stål). *Comp. Biochem. Physiol. C Toxicol. Pharmacol.* **203**, 12–20 (2017).
6. Zhang, Y., Yang, Y., Sun, H. & Liu, Z. Metabolic imidacloprid resistance in the brown planthopper, *Nilaparvata lugens*, relies on multiple P450 enzymes. *Insect Biochem. Mol. Biol.* **79**, 50–56 (2016).
7. Vontas, J. G., Small, G. J., Nikou, D. C., Ranson, H. & Hemingway, J. Purification, molecular cloning and heterologous expression of a glutathione *S*-transferase involved in insecticide resistance from the rice brown planthopper, *Nilaparvata lugens*. *Biochem. J.* **362**, 329–337 (2002).
8. Mao, K. *et al.* Carboxylesterase genes in nitenpyram-resistant brown planthoppers, *Nilaparvata lugens*. *Insect Sci.* **28**, 1049-1060 (2020).
9. Liu, S. *et al.* RNA interference of NADPH-cytochrome P450 reductase of the rice brown planthopper, *Nilaparvata lugens*, increases susceptibility to insecticides. *Pest Manag. Sci.* **71**, 32–39 (2015).

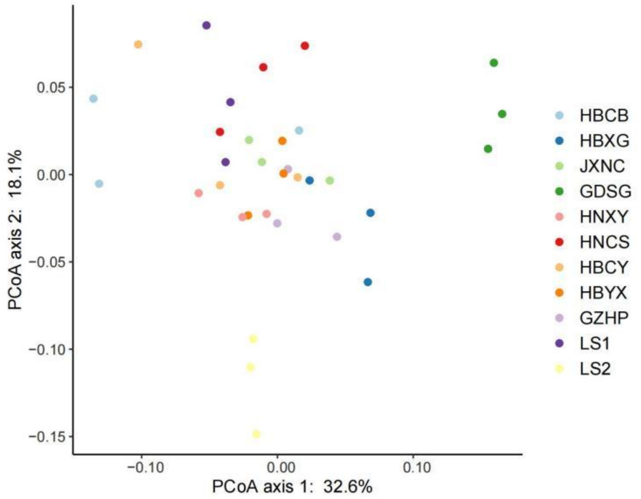

Supplementary Figure 1 Structure difference of transcriptome among different strains base on Bray–Curtis dissimilarity. The color of points indicates different *N. lugens* strains.

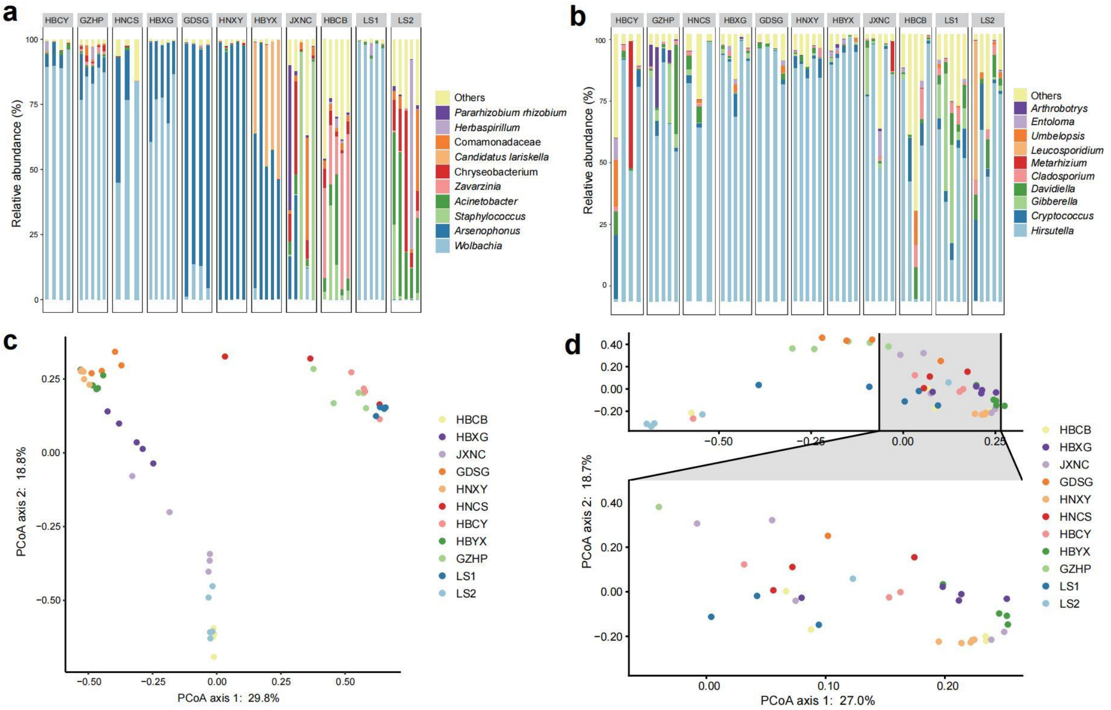

Supplementary Figure 2 Microbiome variation in *Nilaparvata lugens*. a and **b**: Relative abundance of each genus or species of fungi and bacteria, respectively. **c** and **d**: Bray–Curtis based beta diversities of fungi and bacteria communities. The color of points indicates different *N. lugens* strains.

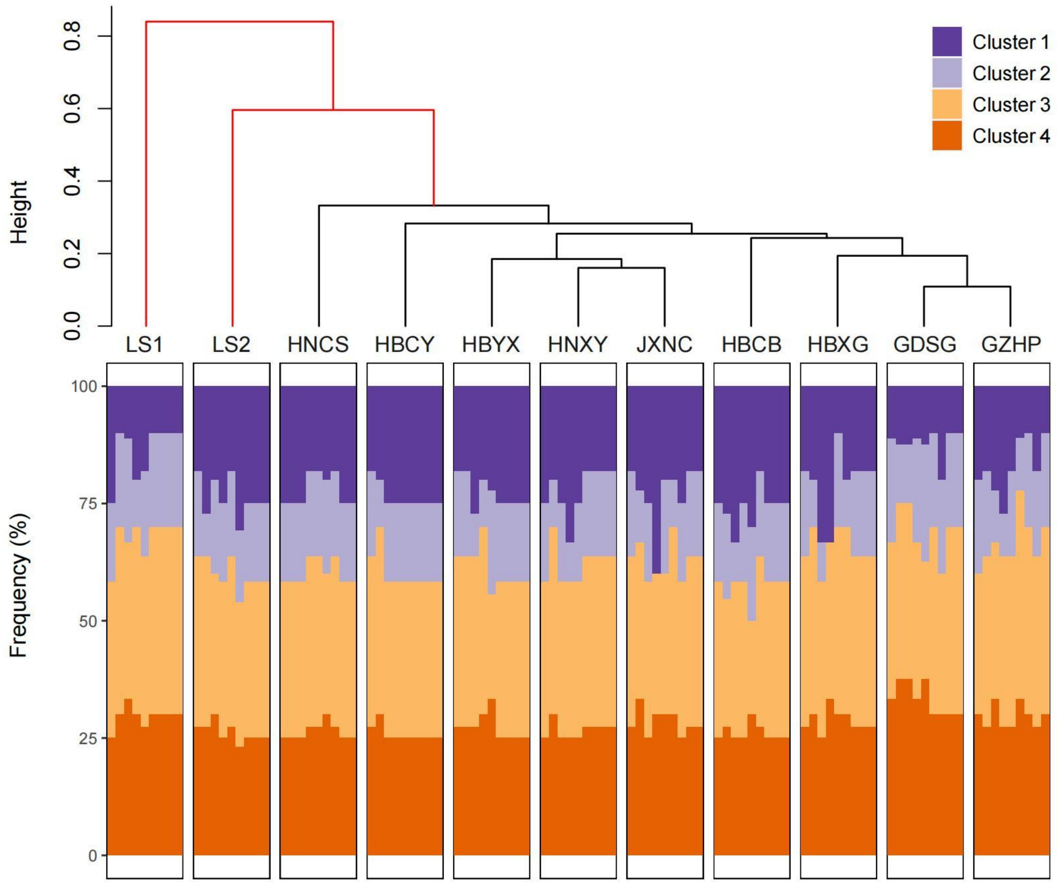

Supplementary Figure 3 The correlation between insecticide susceptibility variation and genetic background. Genetic structure of different *Nilaparvata lugens* strains. Four clusters were defined according to bands with similar frequency that appears in the target segment based on the Bayesian clustering approach. Strains order was defined by the unweighted pair-group method with arithmetic means (UPGMA) tree calculated by genetic distance.
