# Supplementary material for: Microbiome variation correlates with the insecticide susceptibility in different geographic strains of a significant agricultural pest, Nilaparvata lugens
Source: NPJ Biofilms Microbiomes. 2023 Jan 12;9:2. doi: 10.1038/s41522-023-00369-5 (PMC9837087; doi:10.1038/s41522-023-00369-5)
Supplement: Supplementary file 4 — Supplementary Data Caption 1-5 [file 41522_2023_369_MOESM4_ESM.docx]

**Supplementary information**

**Supplementary Information: Supplementary Table 1-19 and Supplementary Figure 1-3.**

**Supplementary Data:**

**Supplementary Dat**a 1 Gene expression profile and function of *Nilaparvata lugens* among different strains.

**Supplementary Data** **2** Interaction networks of LC_50_ value and genes expression.

**Supplementary Data 3** ASV tags and taxonomy of bacteria in *Nilaparvata lugens.*

**Supplementary Data 4** ASV tags and taxonomy of fungi in *Nilaparvata lugens.*

**Supplementary Data 5** Correlations matrix between detoxifying genes expression or LC_50_ and bacterial abundance. If the correlation is not significant (*P* > 0.05), the correlation coefficient was “0”.
